# Trehalose significantly enhances the recovery of serum and serum exosomal miRNA from a paper-based matrix

**DOI:** 10.1038/s41598-017-16960-8

**Published:** 2017-11-30

**Authors:** Shu Hui Neo, Ka Yan Chung, Jia Min Quek, Heng-Phon Too

**Affiliations:** 1 Bioprocessing Technology Institute, Agency for Science, Technology and Research (A*STAR), 20 Biopolis Way, #06-01 Centros, Singapore, 138668 Singapore; 2 Department of Biochemistry, Yong Loo Lin School of Medicine, National University of Singapore, Block MD7, 8 Medical Drive, Singapore, 117597 Singapore

## Abstract

The preservation of nucleic acids from clinical samples is critical to facilitate accurate molecular diagnosis. The use of a paper matrix, Flinders Technology Associates (FTA) Elute cards, to archive DNA and viral RNA is well-documented. However, the feasibility of FTA Elute cards for archiving serum and serum exosomal microRNAs (miRNAs) remains unclear. Here, we performed a comprehensive evaluation of FTA Elute cards for miRNA storage and recovery in different pre-analytical conditions. The recovery of serum miRNA dry-spotted on FTA Elute cards by direct elution with water at high temperature was poor. However, serum miRNAs dry-spotted on the cards were isolated with about 40% yield when using QIAzol lysis reagent and recovery was improved remarkably (>80%) upon extraction from cards pre-treated with trehalose. miRNAs stored on the cards remained stable at room temperature and can be kept for prolonged periods. Furthermore, miRNAs could be similarly recovered from serum exosomes dry-spotted on the cards. Importantly, when using sera from gastric cancer (GC) patients, the miRNAs were efficiently recovered from trehalose pre-treated cards without affecting their representation. Collectively, we have demonstrated the potential of FTA Elute cards to archive serum and serum exosomal miRNAs, making it useful for biomarker discovery and diagnostics.

## Introduction

MicroRNAs (miRNAs) are small non-coding RNAs (19–22 nucleotides) that regulate protein expression and are physiologically significant in several cellular processes^1,2^. Unlike other forms of RNA, miRNAs are remarkably stable in clinical samples like plasma and serum. In these biofluids, miRNAs are protected from degradation by a) encapsulation in microvesicles or exosomes^3^, b) binding to high-density lipoproteins^4^ or c) binding to the Argonaute 2 protein complex (AGO2)^5^. These forms of miRNAs are actively taken by recipient cells^6–10^ and are known to alter biological pathways affecting cellular functions, which highlights a potential role in cellular communication^3,11^. The expressions of certain miRNAs are also known to be altered in different disease states^12^ and hence serve as potential biomarkers for cancer diagnostics and therapies^13,14^.

The availability of high-quality biospecimens is an absolute requirement for biomarker discovery. The quality of the biospecimens depends on a number of pre-analytical variables which includes collection, processing and storage stability^15,16^. The most common and extensively used method of preserving biospecimens is to freeze the samples and store them at low temperatures^17^. In biofluids, miRNAs are stable over a prolonged period of storage when frozen at low temperatures^18–20^. There are however, practical constraints of storing frozen samples and these include capital investments of freezers and space. Furthermore, transportation of these samples to reference laboratories will require costly specialized equipment and conditions^17^.

The Flinders Technology Associates (FTA) Elute cards (GE Healthcare) have been used successfully to collect, transport and archive nucleic acids of various pathogens, which include viral DNA and RNA^21–24^. These cellulose-based cards are chemically treated to lyse cells and trap nucleic acids on the matrix, keeping them stable at room temperature (RT). The nucleic acids can also be eluted easily using water and heat for PCR analysis if required^25^. Hence, they have been extensively used in disease surveillance and pathogen detection^21^.

A recent study described the extraction of miRNAs from dry-spotted plasma on FTA Elute cards using water and heat, a retrieval method recommended by the manufacturer^25,26^. However, we found that serum miRNAs were inefficiently recovered from dry-spotted serum on FTA Elute cards. This prompted us to develop a more reliable method for archiving and retrieving miRNA from serum.

In this study, we recovered serum and serum exosomal miRNAs from FTA Elute cards using a commercially available reagent, QIAzol lysis reagent. This method is efficient without the need to heat the samples. Trehalose is a non-reducing disaccharide sugar commonly used as a cytoprotectant to stabilize proteins^27^, nucleic acids^28,29^ and exosomes^30^. Unexpectedly, we found that FTA Elute cards pre-treated with trehalose significantly enhanced the recovery of these miRNAs.

Overall, we have demonstrated that the FTA Elute cards are suitable to archive serum miRNAs at RT without affecting miRNA representation in expression levels. We have also tested its utility in quantifying miRNAs in dry-spotted gastric cancer (GC) patient serum, suggesting the potential of using FTA Elute cards as a suitable storage medium for serum in clinical studies.

## Results

### Inefficient miRNA extraction from serum-spotted FTA Elute cards using water and heat

**Figure 1:**
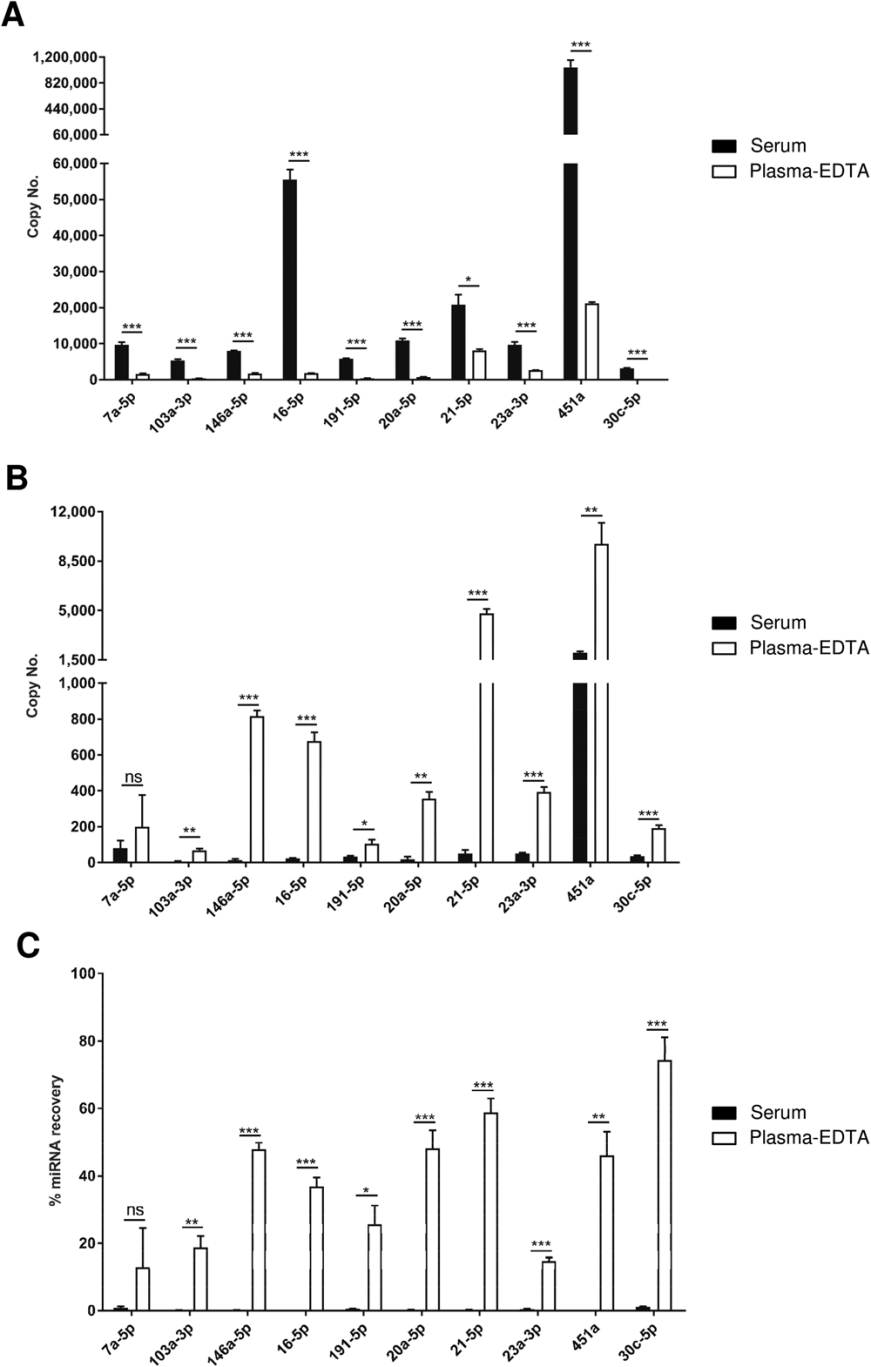
miRNA recovery from serum-spotted FTA Elute cards using water and heat was inefficient. (**A**) RNA was extracted directly from 20 µl serum (black) or 20 µl plasma collected in EDTA-containing tube (plasma-EDTA) (white) using QIAzol lysis reagent. 10 miRNAs were quantified by RT-qPCR based on their copy numbers. RNA was extracted from a 6 mm FTA Elute card disc punch-out spotted with 20 µl serum (black) or 20 µl plasma-EDTA (white), using water and heating at 95 °C for 30 min. 10 miRNAs were quantified by RT-qPCR and their copy numbers (**B**) and % miRNA recovery (**C**) were presented. (% recovery = (Copy number of miRNA extracted from FTA Elute card disc punch-out/Copy number of miRNA extracted directly from serum) × 100%). Each experimental condition was carried out thrice and data were presented as mean ± SEM (***P ≤ 0.001; **P ≤ 0.01; *P ≤ 0.05; ns: not significant, Student’s *t*-test, n = 3).

Total RNA from serum and plasma were first extracted using QIAzol lysis reagent and the expression levels of 10 miRNAs (let-7a-5p, miR-103a-3p, miR-146a-5p, miR-16-5p, miR-191-5p, miR-20a-5p, miR-21-5p, miR-23a-3p, miR-451a, miR-30c-5p) were measured by RT-qPCR. The levels of these miRNAs in serum were found to be higher than in plasma collected using either EDTA (Fig. 1A) or sodium citrate-containing tubes (Supplementary Fig. S1A). To determine extraction efficiency by QIAzol lysis reagent, we spiked in two synthetic single-stranded miRNAs, SP4 and SP9, into serum and plasma. The extraction efficiencies for SP4 and SP9 using QIAzol lysis reagent were >80% (Supplementary Fig. S1B). A recent report has shown the application of water and heat to extract miRNAs from plasma and circulating tumour cells after spotting on FTA Elute cards^26^. To determine if this method could extract miRNAs from serum-spotted FTA Elute cards, serum and plasma samples were dry-spotted and extracted as reported. Unlike plasma, miRNAs were poorly recovered from serum (Fig. 1B,C, Supplementary Fig. S1C and D). It was possible that the 70% ethanol-mediated washing step during water-mediated extraction could have exposed the miRNAs to RNAses. However, our results showed that the recovery of miRNAs from water-mediated extraction remained poor even in the absence of the washing step (Supplementary Fig. S1E).

### miRNA extraction from serum-spotted FTA Elute cards was improved using QIAzol lysis reagent

**Figure 2:**
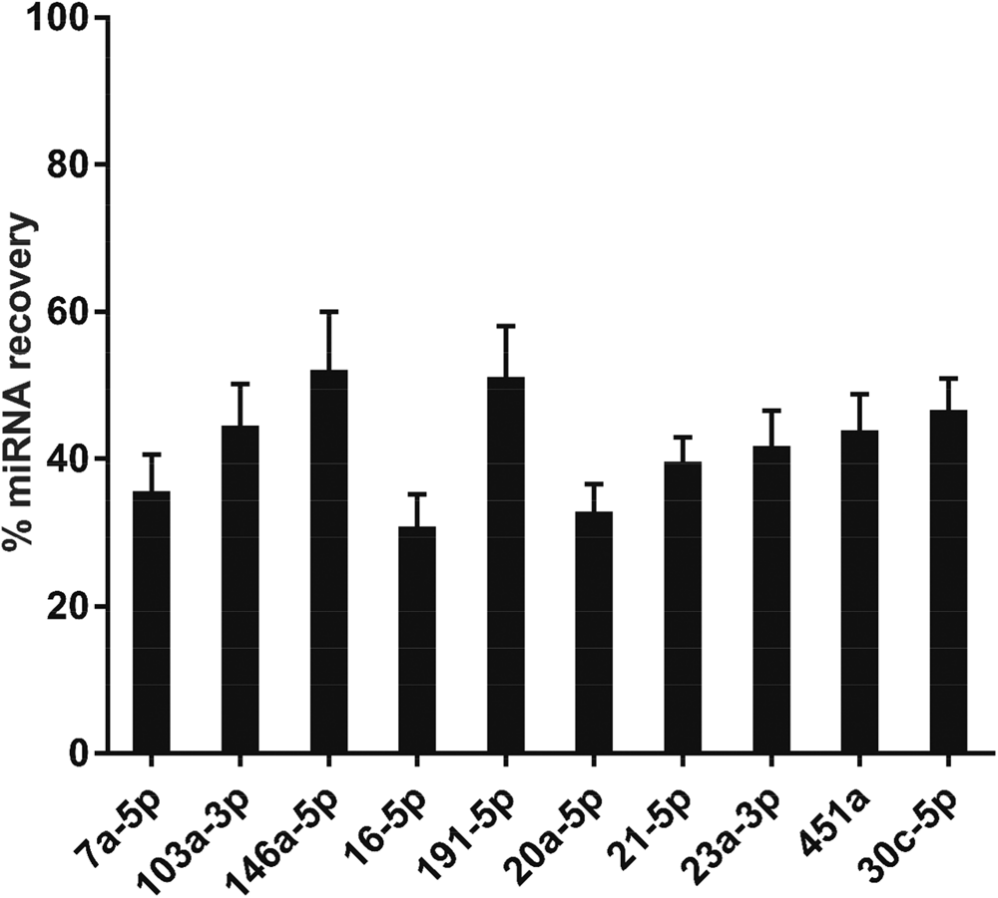
miRNA recovery was achieved from serum-spotted FTA Elute cards using QIAzol lysis reagent. % recovery of each of the 10 miRNAs extracted from a 6 mm FTA Elute card disc punch-out were presented (% recovery = (Copy number of miRNA extracted from FTA Elute card disc punch-out/Copy number of miRNA extracted directly from serum) × 100%). Each experimental condition was carried out thrice and data were presented as mean ± SEM.

Next, an attempt to extract miRNAs from serum dry-spotted on FTA Elute cards was carried out with QIAzol lysis reagent. All 10 miRNAs were recovered with greater efficiency using QIAzol lysis reagent (Fig. 2) as compared to using water and heat (Fig. 1B). The percentage miRNA recovery from FTA Elute cards was about 40% on the average for all 10 miRNAs (Fig. 2). To further investigate whether retrieval of the miRNA may be interfered by the matrix, different concentrations of pure synthetic miRNAs (let-7a-5p and miR-146a-5p) were dry-spotted on FTA Elute cards, followed by miRNA isolation using QIAzol lysis reagent. In parallel, the same concentrations of miRNAs were used directly for RT-qPCR, serving as controls. The result showed that synthetic miRNAs could be efficiently extracted from FTA Elute cards comparable to the controls (Supplementary Fig. S2).

### Improvement of miRNA recovery from FTA Elute cards by trehalose

**Figure 3:**
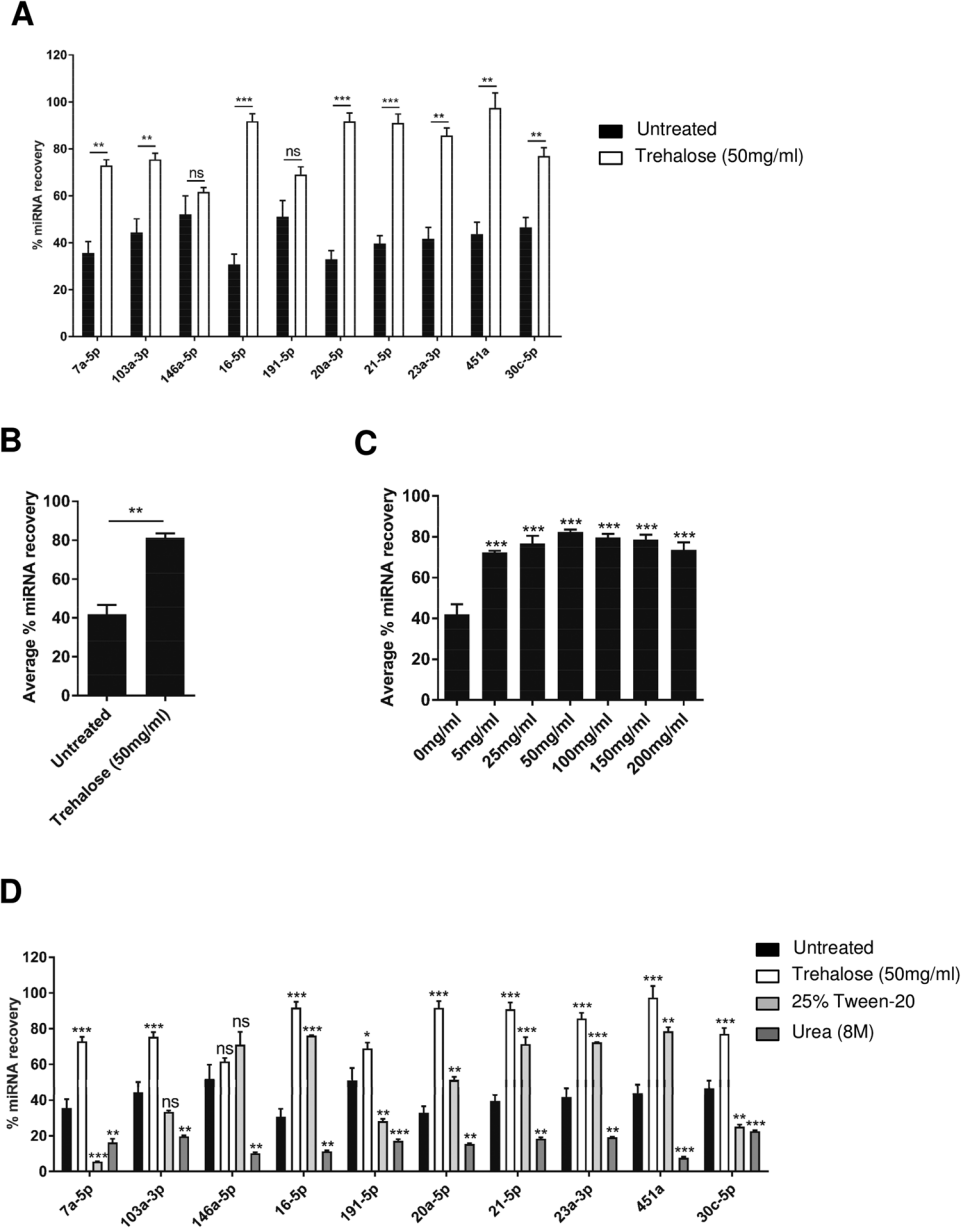
miRNA recovery from serum-spotted FTA Elute cards was improved upon pre-treatment with trehalose. (**A**) 20 µl serum was spotted directly on an untreated (black) or trehalose-treated (white) 6mm FTA Elute card disc punch-out before QIAzol lysis reagent-mediated extraction. 10 miRNAs were quantified and individual % miRNA recovery was calculated (% recovery = (Copy number of miRNA extracted from FTA Elute card disc punch-out/Copy number of miRNA extracted directly from 20 µl serum) × 100%). (**B**) Average % miRNA recovery of all 10 miRNAs was compared between untreated and trehalose-treated FTA Elute card disc punch-out. (**C**) FTA Elute card disc punch-out was pre-treated with different concentration of trehalose before spotting of 20 µl serum. Average % miRNA recovery of all 10 miRNAs was compared between untreated and trehalose-treated FTA Elute card disc punch-out. (**D**) Untreated FTA Elute card disc punch-out (black), trehalose-treated (white), Tween-20 (grey) and Urea (dark grey)-treated FTA Elute card disc punch-out were prepared before spotting with 20 µl serum. For each treatment, 10 miRNAs were quantified and individual % miRNA recovery was compared between untreated and chemically-treated FTA Elute card disc punch-out. Each experimental condition was carried out thrice. Data were presented as mean ± SEM. Statistical analyses were performed using one-way ANOVA analysis followed by Bonferroni’s pairwise comparisons test to compare different treatments with untreated (***P ≤ 0.001; **P ≤ 0.01; *P ≤ 0.05; ns: not significant, n = 3).

To further optimize the miRNA extraction efficiency from FTA Elute cards, the cards were pre-treated with trehalose before spotting of serum samples. Recovery of miRNA from serum dry-spotted on pre-treated FTA Elute cards was unexpectedly greater than with un-treated cards (Fig. 3A). The average miRNA recovery of all 10 miRNAs was significantly enhanced by as much as 2-fold, from ~40% to ~80% (Fig. 3B). We next determined the effect of varying trehalose concentration for FTA Elute cards pre-treatment. There was no dramatic difference with the use of high concentrations of trehalose (>100 mg/ml) (Fig. 3C). Since the highest percentage recovery was observed at 50 mg/ml, subsequent experiments were performed with this concentration. The use of different RNA extraction kits^31–35^ may affect the miRNA recovery from serum dry-spotted on pre-treated FTA Elute cards. To address this, we compared the performance of QIAzol lysis reagent with Trizol-LS reagent, Trizol reagent and column-based RNA isolation kit (Ambion). Our results showed that the average percentage miRNA recovery of all 10 miRNAs across these methods was similar (Supplementary Fig. S3B). We next asked if miRNA enhancement could similarly be achieved using a denaturant (urea) or a surfactant (Tween-20). Pre-treatment with urea resulted in poor miRNA recovery across all 10 miRNAs (Fig. 3D). Interestingly, certain miRNAs were detected from Tween-20 pre-treated FTA Elute cards with recoveries of more than 50% (miR-146a-5p, miR-16-5p, miR-21-5p, miR-23a-3p and miR-451a). Taken together, our data suggests that trehalose is superior in improving miRNA recovery from FTA Elute cards.

### miRNAs stored in FTA Elute cards were stable at room temperature

**Figure 4:**
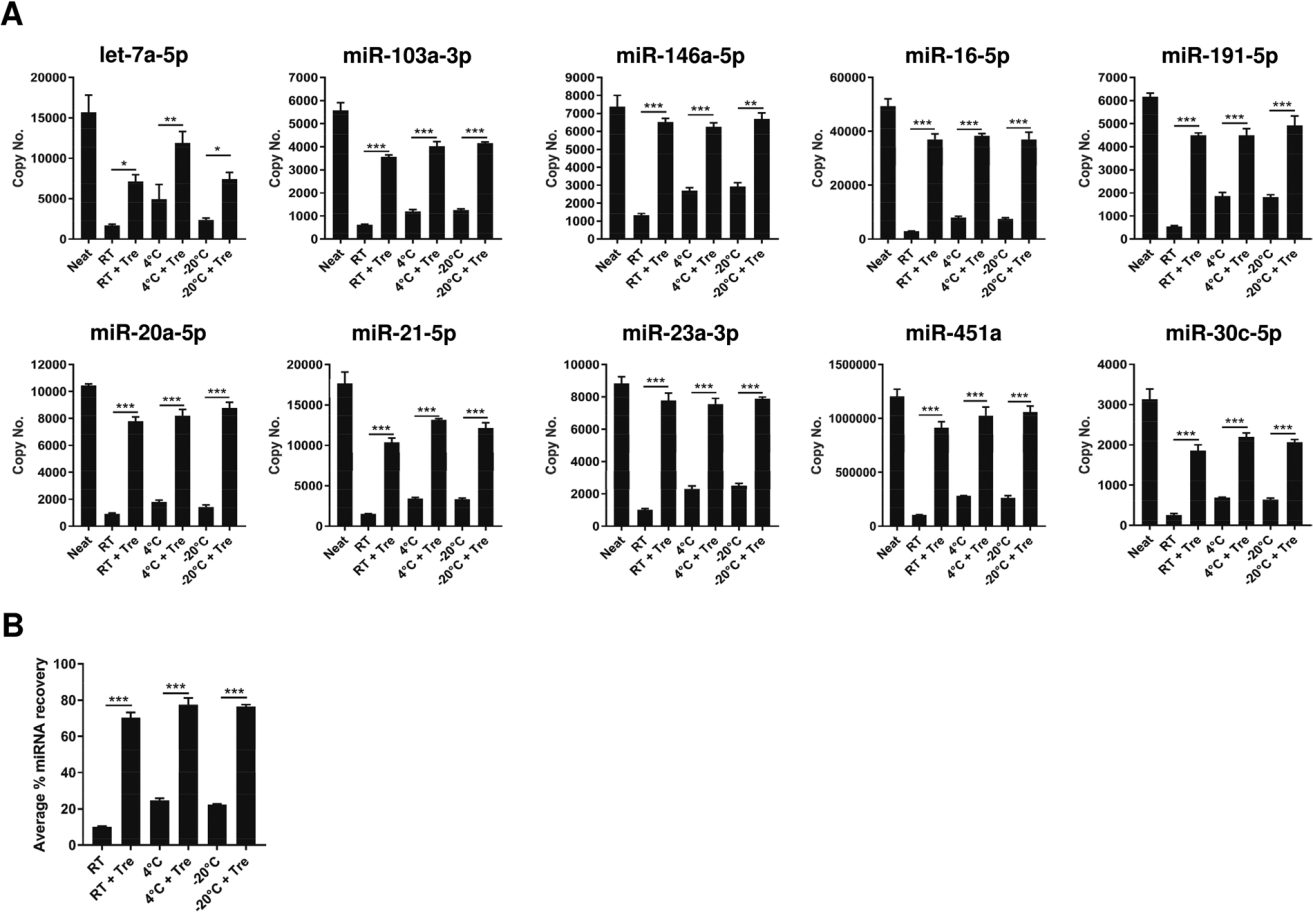
Comparison of different storage temperature and trehalose for FTA Elute card stored for 2 days. (**A**) Untreated or 50 mg/ml trehalose-treated 6 mm FTA Elute card disc punch-out spotted with 20 µl serum were dried and stored at RT, 4 °C or −20 °C for 2 days before RNA was extracted using QIAzol lysis reagent. 10 miRNAs were quantified by RT-qPCR and their copy numbers were presented. Neat refers to copy number of each individual miRNA obtained from 20 µl serum extracted directly using QIAzol lysis reagent. The individual % miRNA recovery was compared between untreated and trehalose-treated FTA Elute card disc punch-out. (**B**) Average % miRNA recovery of all 10 miRNAs was calculated and plotted to compare the effect of pre-treatment of FTA Elute card disc punch-out with 50mg/ml trehalose. Statistical analyses were performed with one-way ANOVA, followed by Bonferroni’s pairwise comparisons test data between selected pairs. Each experimental condition was carried out thrice and data were presented as mean ± SEM (***P ≤ 0.001; **P ≤ 0.01; *P ≤ 0.05, n = 3).

To investigate the storage stability of miRNA on FTA Elute cards, miRNAs were extracted from serum dry-spotted cards that were stored at different temperatures (RT, 4 °C and −20 °C) for 2 days and their respective miRNA yields were compared (Fig. 4A). Regardless of the storage temperature examined, the average recovery of all 10 miRNAs extracted from serum dry-spotted on trehalose pre-treated matrix was similar (Fig. 4B). In addition, we observed similar trend when these serum dry-spotted on pre-treated FTA Elute cards were kept for 1 week (Supplementary Fig. S4) and 2 weeks (Supplementary Fig. S5).

### Long term storage of FTA Elute cards did not affect miRNA representation

**Figure 5:**
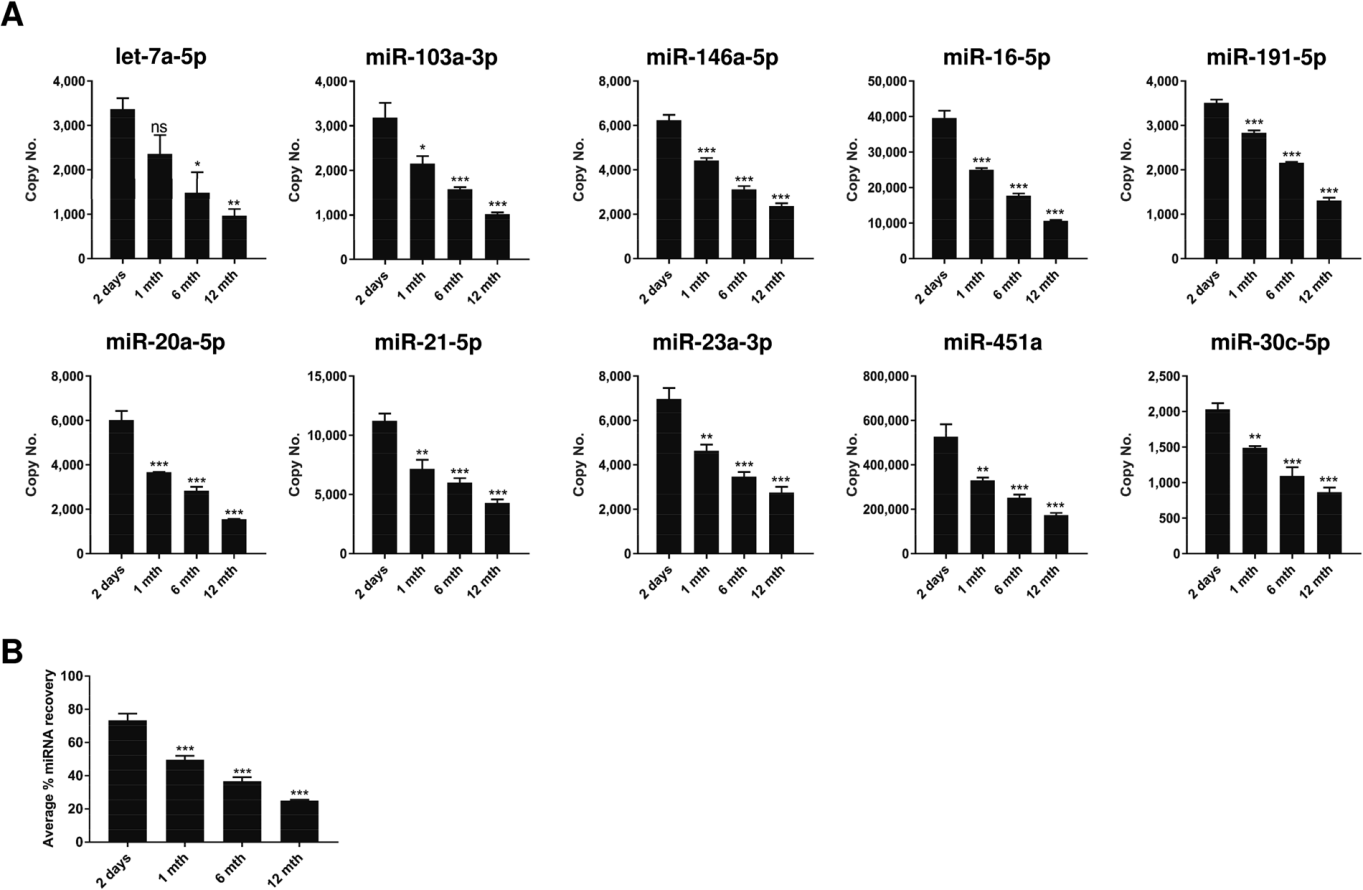
Effect on miRNA yield during long-term FTA Elute card storage. (**A**) 20 µl serum spotted on trehalose-treated 6 mm FTA Elute card disc punch-out was subjected to accelerated aging testing of 1, 6 and 12 months before RNA was extracted using QIAzol lysis reagent. 10 miRNAs were quantified. The copy number of each miRNA was compared against the copy number obtained after storage at RT for 2 days. (**B**) Average % miRNA recovery of all 10 miRNAs for accelerated aging of 1, 6, 12 months was determined and compared with 2 days. Statistical analyses were performed with one-way ANOVA, followed by Bonferroni’s pairwise comparisons test data. Each experimental condition was carried out thrice and data were presented as mean ± SEM (***P ≤ 0.001; **P ≤ 0.01; *P ≤ 0.05; ns: not significant, n = 3).

Depending on the intended use, it may be necessary to store sample dry-spotted on FTA Elute cards for prolonged periods before the contents are analysed. The potential of FTA Elute cards as a device for archiving and long-term storage of miRNA was assessed next. Serum was dry-spotted on trehalose pre-treated FTA Elute cards and aged by thermal acceleration to the equivalence of 1, 6 and 12 months^36^. The sample was then compared with equivalent serum dry-spotted on trehalose pre-treated matrix for 2 days at RT. As shown in Fig. 5A, prolonged storage showed a gradual decrease in the amount of miRNA recovered. After 12 month of accelerated aging, the average percentage miRNA recovery was reduced from ~80% to ~25% (Fig. 5B). It is important to note that although miRNA yield was significantly decreased upon storage, miRNA representation in expression levels remained unchanged (Supplementary Fig. S6).

### Serum exosomal miRNAs could be extracted from FTA Elute cards

**Figure 6:**
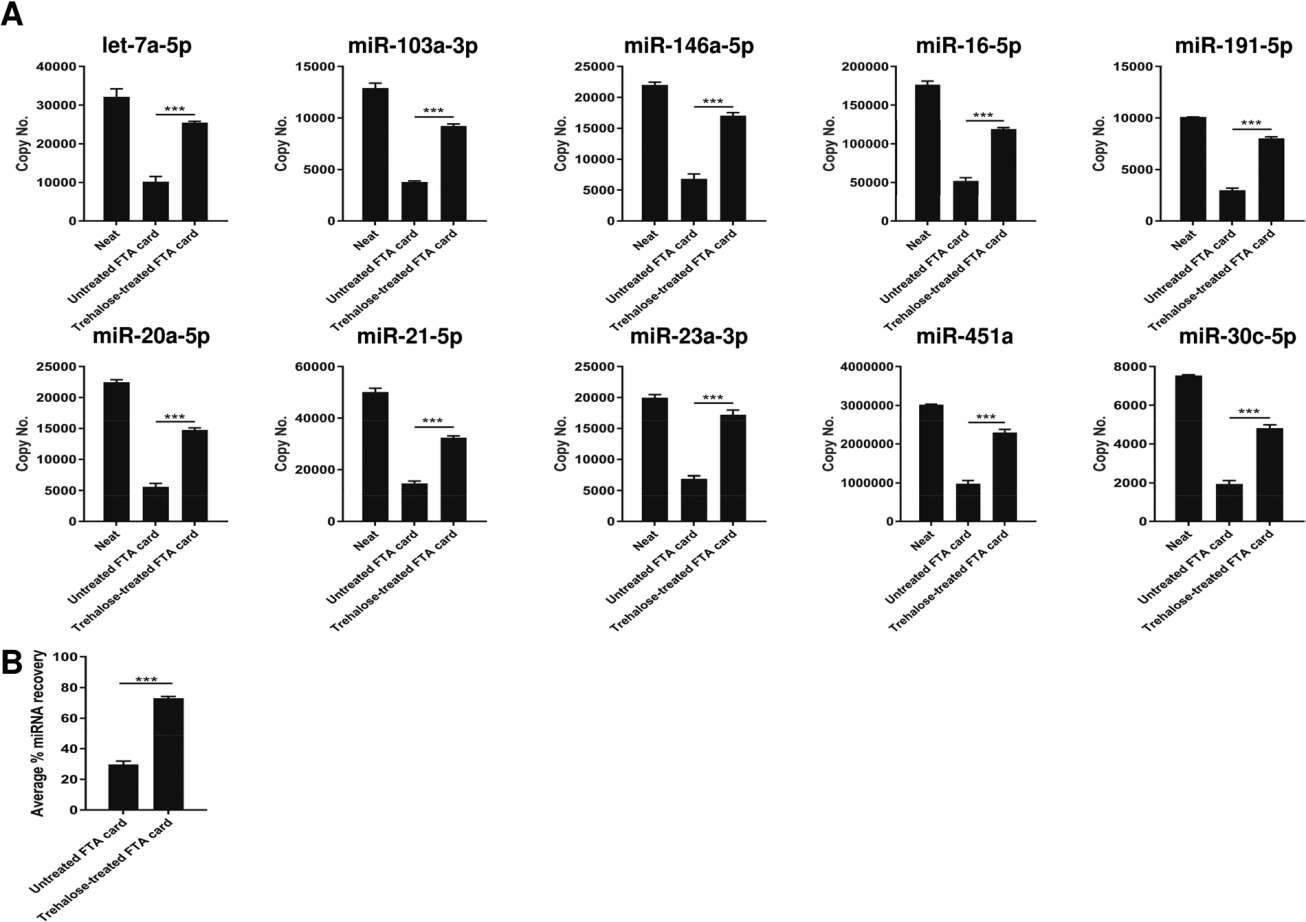
Extraction of serum exosomal miRNAs from FTA Elute card. (**A**) 20 µl serum exosomes isolated using precipitation reagent was spotted directly on untreated or trehalose-treated FTA Elute card disc punch-out. 10 miRNAs were quantified and their copy numbers were compared. Neat refers to copy number of each individual miRNA obtained from 20 µl exosomes extracted directly using QIAzol lysis reagent. Comparison was made between copy numbers of individual miRNA recovered from untreated and trehalose-treated FTA Elute card. (**B**) Average % miRNA recovery of all 10 miRNAs was calculated and plotted. Statistical analyses were performed with one-way ANOVA, followed by Bonferroni’s pairwise comparisons test data for selected pairs. Each experimental condition was carried out thrice and data were presented as mean ± SEM (***P ≤ 0.001, n = 3).

We next investigated the ability of FTA Elute cards to archive and subsequently retrieve serum exosomal miRNAs. miRNAs recovered from dry-spotted serum exosomes on FTA Elute cards using QIAzol lysis reagent were compared with miRNAs extracted directly from serum exosomes, serving as controls (neat). The amount of miRNAs recovered from serum exosomes dry-spotted on untreated FTA Elute cards was poor (Fig. 6A). However, with trehalose pre-treated cards, >2-fold increase in miRNA recovery was observed, indicating that trehalose was useful in improving miRNA recovery of serum exosomes dry-spotted on FTA Elute cards (Fig. 6B).

### Storage on FTA Elute cards did not bias serum miRNA representation with GC serum

**Figure 7:**
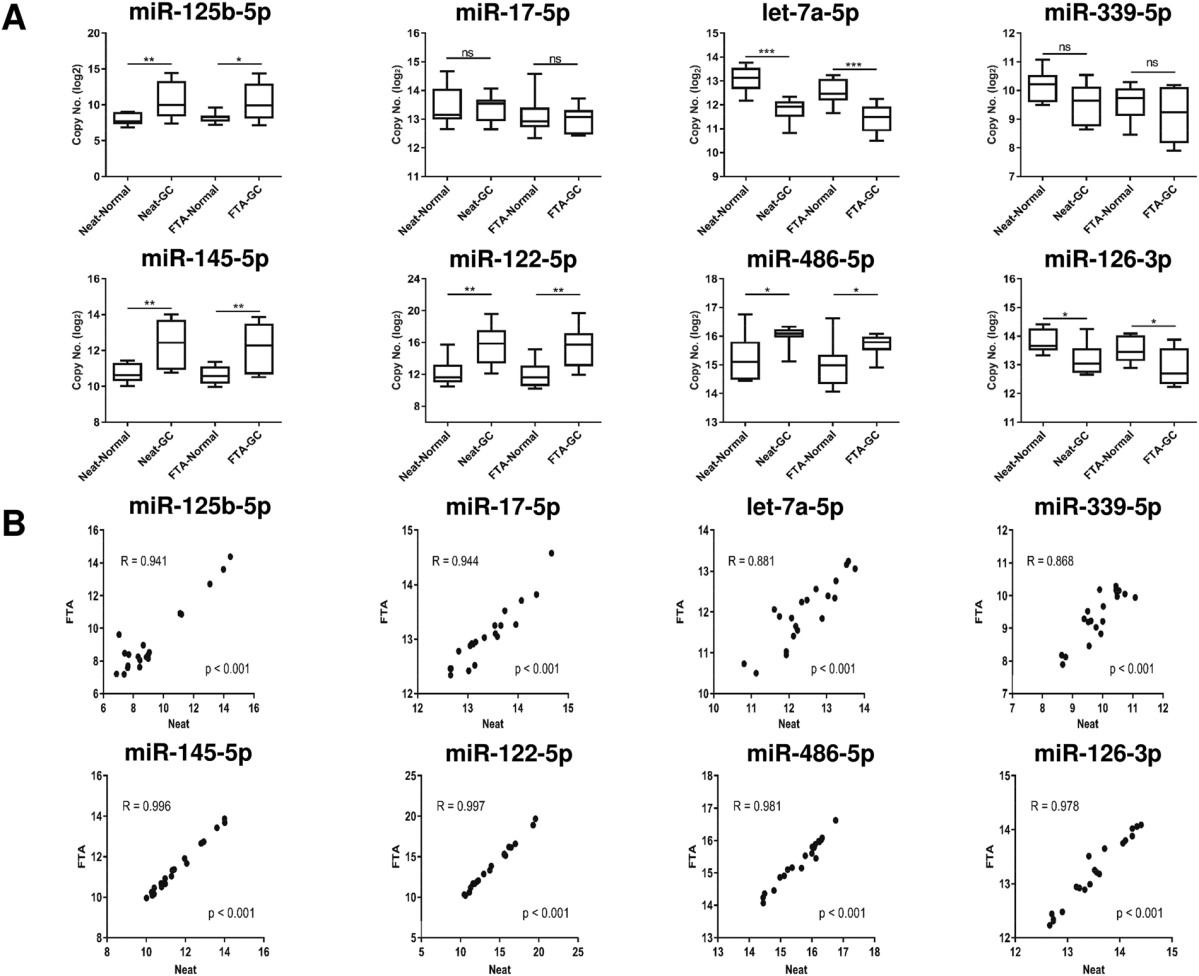
miRNA expression level in GC patient and paired controls. (**A**) 20 µl of each of the 10 GC patients and 10 paired control serum were spotted on individual 6 mm FTA Elute card disc punch-out. 8 miRNAs were quantified and miRNA recovery was compared with the sample in which RNA was isolated directly from 20 µl serum using QIAzol lysis reagent (neat). (**B**) Correlation of miRNA representation from neat serum or FTA Elute card discs. Each experimental condition consisted of 10 biological replicates and data were presented as mean ± SEM. Statistical analyses were performed with one-way ANOVA, followed by Bonferroni’s pairwise comparisons test data for selected pairs (***P ≤ 0.001; **P ≤ 0.01; *P ≤ 0.05; ns: not significant).

To test the feasibility of FTA Elute cards for storage of clinical samples for the detection of differentially expressed miRNAs between normal and cancer patient, 10 GC sera and paired controls were dry-spotted on trehalose pre-treated FTA Elute cards. In parallel, miRNAs from equivalent amount of serums were extracted directly, serving as controls (neat). Eight miRNAs (miR-125-5p, miR-17-5p, let-7a-5p, miR-339-5p, miR-145-5p, miR-122-5p, miR-486-5p and miR-126-3p) known to be differentially expressed between GC and normal individuals were assayed. Our results demonstrated that miRNA recovered from trehalose pre-treated cards did not bias serum miRNA representation (Fig. 7A), with correlation of >0.86 (Fig. 7B).

## Discussion

Although FTA Elute cards have been successfully used for the collection and storage of viral RNA, and DNA from biological samples^21–24^, there are instances where the recovery of nucleic acids was unacceptably low, as with enteroviral RNA (~6.1%)^24^. Hence, in this study, we investigated the extraction efficiency of serum and serum exosomal miRNAs dry-spotted on FTA Elute cards and its utility for prolonged storage of miRNAs on the paper matrix.

We first evaluated the recovery of serum miRNAs dry-spotted on FTA Elute cards by following a recent study which highlighted the convenience of using water and heat to directly extract miRNA from plasma and circulating tumor cells spotted on the paper matrix^26^. However, we found that the recovery of the miRNAs from serum dry-spotted on the same matrix using the reported protocol was consistently poor, despite the higher abundance of the 10 miRNAs in serum as compared to plasma (Fig. 1A). The miRNAs analysed in our study are present at various levels in the serum. Using QIAzol lysis reagent, we were able to extract miRNAs from the FTA Elute cards with sufficient efficiencies (Fig. 2). The processing procedures used for serum and plasma preparations may have contributed to this difference in the recovery of miRNAs from serum and plasma dry-spotted on FTA Elute cards.

There are several kits available in the market for miRNA extraction from biological samples, which can be broadly classified into two groups: a) phenol:chloroform-based extraction; b) pure column-based technique with the use of a phenol-free lysis buffer and column for RNA recovery. Several studies have compared and showed the differences in the yield and quality of miRNA obtained from these RNA extraction methods^31–35,37^. We found that these methods were compatible with the use of trehalose pre-treated FTA Elute cards as the average recovery of all 10 miRNAs extracted from serum dry-spotted on trehalose pre-treated matrix was similar, hence providing alternatives to the use of QIAzol lysis reagent (Supplementary Fig. S3B).

Trehalose, a non-reducing disaccharide, is known to increase the stability of biomolecules like enzymes and nucleic acids in their dry state^38–40^. We found that the percentage recovery of the miRNAs increased (~80%) when extracted with QIAzol lysis reagent from trehalose but not from urea or Tween-20 pre-treated FTA Elute cards (Fig. 3D). It is likely that trehalose binds to the negatively charged phosphates in nucleic acids to displace water molecules and potentially protects them from hydrolysis and oxidation. This then decreases their structural conformations and enhances their chemical stability^28,41^. It remains to be determined whether pre-treatment of FTA Elute cards with these chemicals may have affected downstream procedures when quantifying miRNAs by RT-qPCR.

It was reported that the storage of serum at RT resulted in significant reduction of miRNA levels as compared with storage at −80 °C, but there was no difference between storage at −80 °C and −20 °C^18^. Upon deposition of serum onto FTA Elute cards and its subsequent storage in RT, 4 °C and −20 °C for 2 days, there was no significant difference in the amount of miRNAs recovered. However, the recovery of serum miRNAs was much higher (Fig. 4) with serum dry-spotted on trehalose pre-treated FTA Elute cards kept at these storage temperatures. With prolonged storage, the average percentage miRNA recovery from FTA Elute cards was reduced overtime (Fig. 5B) but there was no change to the miRNA representation in expression levels of the miRNAs examined (Supplementary Fig. S6). This suggests the potential use of FTA Elute cards in disease surveillance when such studies are commonly carried out over prolonged periods. For long term storage, pre-amplification steps can be considered after reverse-transcription^42^ to compensate for the reduced miRNA recovery, especially for miRNAs expressed at low levels.

The development of protocols for preserving nucleic acids in clinical specimens is critical for accurate molecular testing. In our study, we investigated the expression levels of eight miRNAs (miR-125-5p, miR-17-5p, let-7a-5p, miR-339-5p, miR-145-5p, miR-122-5p, miR-486-5p and miR-126-3p)^43–51^ that have been identified as potential biomarkers for GC detection. The subsequent extraction of these miRNAs from serum dry-spotted on FTA Elute cards showed comparable differential expressions of these miRNAs between normal and GC serum (Fig. 7). We have also demonstrated a previously unreported use of FTA Elute card in miRNA extraction from dry-spotted serum exosomes (Fig. 6), marking a potential for paper matrix to store and detect serum exosomal miRNAs.

In conclusion, we have developed a method using QIAzol lysis reagent and trehalose pre-treated FTA Elute card to archive and retrieve miRNAs from serum or serum exosomes dry-spotted on a paper matrix where the representation in expression levels is retained quantitatively. This method can hence be easily incorporated into clinical studies, facilitating transport of specimens across laboratories for diagnostics.

## Methods

### Plasma/Serum samples

Pooled normal human plasma, collected in EDTA vial (IPLA-N) and pooled normal human serum (IPLA-SER) were purchased from Innovative Research. Human plasma collected with sodium citrate (P9523) was purchased from Sigma. Serum samples of 10 healthy controls and 10 gastric cancer patients were purchased from Asterand Bioscience.

### Direct isolation of serum/plasma RNA by QIAzol lysis reagent

Total RNA from serum/plasma was isolated using miRNeasy serum/plasma isolation kit (Qiagen) according to manufacturer’s protocol. Briefly, 20 µl serum/plasma was added with 180 µl phosphate-buffered saline (PBS). 1 ml QIAzol lysis reagent was spiked with a) 1 µg MS2 carrier RNA (Roche) to reduce loss of RNA during isolation and b) a set of 3 synthetic small RNAs (MiRXES, Singapore) with sequences distinct from any of the 2588 annotated mature human miRNAs (miRBase version 21) to monitor RNA isolation efficiency. This QIAzol mixture was aliquoted to the 200 µl sample for lysis. 200 µl chloroform was then added, thoroughly mixed and centrifuged at 18,000 g for 15 minutes (min). 600 µl aqueous phase was subsequently removed for spin-column based purification and RNA was eluted in 30 µl nuclease-free water.

### FTA Elute card extraction of miRNAs from serum/plasma by QIAzol lysis reagent

20 µl serum/plasma was applied to a 6 mm FTA Elute card disc punch-out (GE Healthcare Life Sciences) and dried at RT for at least 3 hours (hr). The disc was then placed in 1.5 ml centrifuge tube and added with 180 µl PBS. QIAzol mixture was then added to the 200 µl sample for lysis and processed similarly as described above.

### FTA Elute card extraction of miRNAs from serum/plasma by water elution

The application of plasma on FTA Elute cards was performed as described previously^26^. Briefly, 20 µl serum/plasma was spotted directly onto a 6 mm FTA Elute card disc punch-out and dried at RT for at least 3 hr. Subsequently, the disc was washed in 500 µl 70% ethanol, dried and soaked in 30 µl nuclease-free water. miRNAs were then eluted at 95 °C for 30 min.

### FTA Elute card extraction of miRNAs from serum by Trizol reagent and Trizol-LS reagent

20 µl serum was spotted directly onto a 6 mm FTA Elute card disc punch-out and dried at RT for at least 3 hr. Total RNA from serum was isolated using Trizol reagent or Trizol-LS reagent according to manufacturer’s protocol with minor modifications. The disc was placed in 1.5 ml centrifuge tube and added with 180 µl PBS. The isolation procedure was similarly performed as described above with the use of QIAzol lysis reagent, except that centrifugation was performed at 12,000 g for 15 min at 4 °C. 600 µl aqueous phase was subsequently aliquoted into new tube and supplemented with 4 µl linear acrylamide and 500 µl isopropanol for RNA precipitation. The RNA pellet was washed with 75% ethanol and reconstituted in 30 µl nuclease-free water.

### FTA Elute card extraction of miRNAs from serum by column-based kit

20 µl serum was spotted directly onto a 6 mm FTA Elute card disc punch-out and dried at RT for at least 3 hr. Total RNA from serum was isolated using RecoverAll Total Nucleic Acid Isolation Kit (Ambion) according to manufacturer’s protocol.

### Chemical treatment of FTA Elute card

D-(+)-Trehalose (Sigma), Tween 20 (Sigma) and Urea (Sigma) was individually dissolved in nuclease-free water to specific concentrations. A 6 mm FTA Elute card disc punch-out was immersed in these solutions for 10 min and dried at RT for 30 min. Subsequently, 20 µl serum was applied to the pre-treated disc, dried at RT for at least 3 hr and processed by QIAzol lysis reagent as described above.

### Storage conditions

20 µl serum was spotted directly onto a 6 mm FTA Elute card disc punch-out that was pre-treated with or without trehalose solution (50 mg/ml). The discs were dried at RT for at least 3 hr before transferring them into ziplock bags containing desiccants. These samples were stored at 3 different temperatures: −20 °C, 4 °C and RT for 2 days, 1 week and 2 weeks. To study long-term storage, samples were stored at 60 °C for 3, 14 and 28 days to simulate accelerated aging for 1, 6 and 12 months respectively, based on Q10 temperature coefficient (http://lso-inc.com/medical-package-testing/accelerated-aging.html). After the indicated time-points, the discs were processed by QIAzol lysis reagent for RNA isolation.

### Isolation of serum exosomes using polymer-based precipitation

Serum was precleared by centrifugation at 2,000 g for 20 min followed by 10,000 g for 30 min. Exosomes were then isolated from 200 µl pre-cleared serum using Total Exosome Isolation Reagent (from serum) (Invitrogen) according to manufacturer’s protocol. The exosome pellet was resuspended in 200 µl PBS for direct RNA extraction (Neat) using QIAzol lysis reagent. For spotting onto a 6 mm FTA Elute card disc punch-out, the pellet was resuspended in 20 µl PBS, applied to the disc and processed by QIAzol lysis reagent.

### RNA extraction efficiency

To determine RNA extraction efficiency using QIAzol lysis reagent, serum/plasma were spiked with two synthetic single-stranded RNAs, at 10^8^ copies of SP4 and at 10^8^ copies of SP9 (MiRXES) and processed using QIAzol lysis reagent. Sequences of SP4 and SP9 are distinct from any of the 2588 annotated mature human miRNAs (miRBase version 21). The same amount of SP4 and SP9 was used directly for reverse transcription and qPCR for comparison.

Synthetic miRNAs let-7a-5p and miR-146a-5p (Sigma) were serially diluted from a concentration of 10^8^ copies/µl to 10^4^ copies/µl. 1 µl synthetic let-7a-5p or miR-146a-5p were spotted directly onto a 6 mm FTA Elute card disc punch-out and dried at RT for at least 3 hr. Subsequently, the disc was processed using QIAzol lysis reagent. The efficiency of miRNA extraction from the disc was assessed by comparing result derived from synthetic miRNAs used directly for reverse transcription-and qPCR.

### Reverse transcription

RNA was reverse-transcribed using ImProm-II Reverse Transcription System (Promega) with specific primers for the 17 miRNA targets (let-7a-5p, miR-103a-3p, miR-146a-5p, miR-16-5p, miR-191-5p, miR-20a-5p, miR-21-5p, miR-23a-3p, miR-451a, miR-30c-5p, miR-125b-5p, miR-17-5p, miR-339-5p, miR-145-5p, miR-122-5p, miR-486-5p and miR-126-3p) and 3 exogenous spike-in controls (MiRXES). cDNA synthesis was carried out in a total reaction volume of 15 µl containing 5 µl RNA, 1X ID3EAL miRNA RT buffer, enzyme and 1X ID3EAL miRNA specific primers (MiRXES). The reaction mixture was incubated at 42 °C for 30 min followed by 90 °C for 5 min to inactivate the reverse transcriptase on C1000 Touch Thermal Cycler (Biorad). Six tenfold serial dilution of synthetic miRNAs were reversed transcribed with the samples for the determination of miRNA copy numbers from the same microplate.

### Real time-quantitative PCR (RT-qPCR)

qPCR reaction was carried out using ID3EAL miRNA qPCR reagents (MiRXES) with specific primer pairs for each of the 17 miRNA targets and 3 exogenous spike-in controls. Each cDNA sample was diluted 10 times with water and added in duplicates onto a 384 well plate (AB systems). PCR amplification was carried out in a total reaction volume of 15 µl containing 5 µl diluted cDNA, 1X ID3EAL miRNA qPCR master mix, 1X ID3EAL miRNA qPCR assay reagents (MiRXES), topped up with nuclease-free water. qPCR amplification and detection were performed on QuantStudio 5 Real-Time PCR System (Thermo Scientific) with the following cycling conditions: 95 °C for 10 min, 40 °C for 5 min, followed by 40 cycles of 95 °C for 10 sec and 60 °C for 30 sec (optical reading). Raw cycles to threshold (Ct) values were calculated using QuantStudio Design & Analysis Software 1.4.1 with automatic baseline setting and a threshold of 0.4.

### Data processing

Technical variations during RNA isolation were first normalized by the 3 exogenous spike-in controls. Ct values were then processed and the absolute copy numbers of the target miRNAs in each sample were determined through intra-polation of the synthetic miRNA standard curves. Data are presented as the mean ± standard error of mean (SEM) and are representative of at least three independent experiments. Statistical analyses for comparison between two groups were performed with two-tailed unpaired student’s *t*-test. Statistical analyses for comparison among three or more groups were performed with one-way ANOVA followed by Bonferroni’s pairwise comparisons test for selected pairs. All statistical analyses were performed using GraphPad Prism 7.0 (GraphPad Software).

### Data availability statement

All relevant data are within the paper and its Supplementary Information files.

## Electronic supplementary material

Supplementary Information
