# Supplementary material for: Trehalose significantly enhances the recovery of serum and serum exosomal miRNA from a paper-based matrix
Source: Sci Rep. 2017 Nov 30;7:16686. doi: 10.1038/s41598-017-16960-8 (PMC5709463; doi:10.1038/s41598-017-16960-8)

Trehalose significantly enhances the recovery of serum and serum exosomal miRNA from a paper-based matrix

Shu Hui Neo[1,*], Ka Yan Chung[1], Jia Min Quek[1], Heng-Phon Too[1,2,*]

[1]Bioprocessing Technology Institute, Agency for Science, Technology and Research (A*STAR), 20 Biopolis Way, #06-01 Centros, Singapore, 138668, Singapore.

[2]Department of Biochemistry, Yong Loo Lin School of Medicine, National University of Singapore, Block MD7, 8 Medical Drive, Singapore, 117597 Singapore.

*Correspondence:

Heng-Phon Too heng-phon_too@nuhs.edu.sg

Shu Hui Neo neo_shu_hui@bti.a-star.edu.sg

Supplementary Information

Supplementary Figure 1. miRNA recovery from serum-spotted FTA Elute card was lower than plasma collected in sodium citrate-collection tube when extracted using water and heat. **(A)** RNA was extracted directly from 20 µl serum (black) or 20 µl plasma collected in sodium citrate-containing tube (plasma-citrate) (white) using QIAzol lysis reagent. 10 miRNAs in each sample were quantified by RT-qPCR based on their copy numbers. **(B)** Two synthetic miRNAs (SP4 and SP9) were spiked into 20 µl serum or plasma collected using either EDTA- or sodium citrate-collection tubes and RNA was extracted directly using QIAzol lysis reagent. %

recovery of these two miRNAs was determined by comparing the copy numbers obtained from same amount of synthetic miRNAs used directly for RT-qPCR. RNA was extracted from a 6mm FTA Elute card disc punch-out spotted with 20 µl serum (black) or 20 µl plasma-citrate (white), using water and heating at 95°C for 30 min. 10 miRNAs were quantified and their copy numbers **(C)** and individual % miRNA recovery **(D)** were presented. **(E)** RNA was extracted from a 6mm FTA Elute card disc punch-out spotted with 20 µl serum using either water and heating at 95°C for 30 min (Water) or QIAzol lysis reagent (QIAzol). These two methods were performed in absence (-Washing) or presence of washing (+ Washing) with 70% ethanol. (% recovery = (Copy number of miRNA extracted from FTA Elute discs/ Copy number of miRNA extracted directly from serum) x 100%). Student's *t*-test was performed for (A, C, D). One-way ANOVA analysis, followed by Bonferroni's pairwise comparisons test data was performed for (E). Each experimental condition was carried out thrice and data were presented as mean ± SEM (*** P≤0.001; ** P≤0.01; ns: not significant, n=3).

Supplementary Figure 2. Extraction of synthetic miRNAs from FTA Elute cards. Synthetic miRNA let-7a-5p **(A)** and miR-146a-5p **(B)** were spotted on FTA Elute card disc punch-out at different concentrations. RNA was then isolated from FTA Elute card disc punch-out (grey) using QIAzol lysis reagent and compared with pure synthetic miRNA (neat) used directly for RT-qPCR (black). Each experimental condition was carried out in triplicates. Graph indicates Ct value with SEM obtained at each miRNA concentration.

Supplementary Figure 3. miRNA recovery from serum-spotted trehalose pre-treated FTA Elute card using phenol-based extraction or column-based

extraction were similar. (A) 20 µl serum spotted on trehalose-treated 6mm FTA Elute card disc punch-out was subjected to RNA extraction using phenol-containing reagents (QIAzol lysis reagent, Trizol reagent, Trizol-LS reagent) or column-based RNA extraction kit (Ambion).10 miRNAs were quantified and individual % miRNA recovery of each method was calculated (% recovery = (Copy number of miRNA extracted from FTA Elute card disc punch-out / Copy number of miRNA extracted directly from 20 µl serum) x 100%). **(B)** Average % miRNA recovery of all 10 miRNAs was determined. One-way ANOVA analysis, followed by Bonferroni's pairwise comparisons test, was performed to compare other methods with QIAzol lysis reagent. Each experimental condition was carried out thrice and data were presented as mean ± SEM (*** $P \leq 0.001$; ** $P \leq 0.01$; ns: not significant, n=3).

Supplementary Figure 4. Comparison of different storage temperature and trehalose for FTA Elute card stored for 1 week. **(A)** Untreated or 50mg/ml trehalose-treated 6mm FTA Elute card disc punch-out spotted with 20ul serum were dried and stored at RT, 4°C or -20°C for 1 week before RNA was extracted using QIAzol lysis reagent. 10 miRNAs were quantified by RT-qPCR and their copy numbers were presented. Neat refers to copy number of each individual miRNA obtained from 20 µl serum extracted directly using QIAzol lysis reagent. The individual % miRNA recovery was compared between untreated and trehalose-treated FTA Elute card disc punch-out. **(B)** Average % miRNA recovery of all 10 miRNAs was calculated and plotted to compare the effect of pre-treatment of FTA Elute card disc punch-out with 50mg/ml trehalose. Statistical analyses were performed with one-way ANOVA, followed by Bonferroni's pairwise comparisons test

data between selected pairs. Each experimental condition was carried out thrice and data were presented as mean ± SEM (*** *P*≤0.001; ** P≤0.01; * *P*≤0.05, n=3).

Supplementary Figure 5. Comparison of different storage temperature and trehalose for FTA Elute card stored for 2 weeks. **(A)** Untreated or 50mg/ml trehalose-treated 6mm FTA Elute card disc punch-out spotted with 20ul serum were dried and stored at RT, 4°C or -20°C for 2 weeks before RNA was extracted using QIAzol lysis reagent. 10 miRNAs were quantified by RT-qPCR and their copy numbers were presented. Neat refers to copy number of each individual miRNA obtained from 20 µl serum extracted directly using QIAzol lysis reagent. The individual % miRNA recovery was compared between untreated and trehalose-treated FTA Elute card disc punch-out. **(B)** Average % miRNA recovery of all 10 miRNAs was calculated and plotted to compare the effect of pre-treatment of FTA Elute card disc punch-out with 50mg/ml trehalose. Statistical analyses were performed with one-way ANOVA, followed by Bonferroni's pairwise comparisons test data between selected pairs. Each experimental condition was carried out thrice and data were presented as mean ± SEM (*** *P*≤0.001; ** P≤0.01; * *P*≤0.05, n=3).

Supplementary Figure 6. miRNA representation in long-term storage samples. 20 µl serum spotted on trehalose-treated 6mm FTA Elute card disc punch-out was subjected to accelerated aging of 1, 6 and 12 months before RNA was extracted using QIAzol lysis reagent. 10 miRNAs were quantified and Ct values were compared with 20 µl serum extracted directly using QIAzol lysis reagent (neat). Each experimental condition was carried out in thrice and data were presented as mean Ct ± SEM.

Supplementary Figure 1

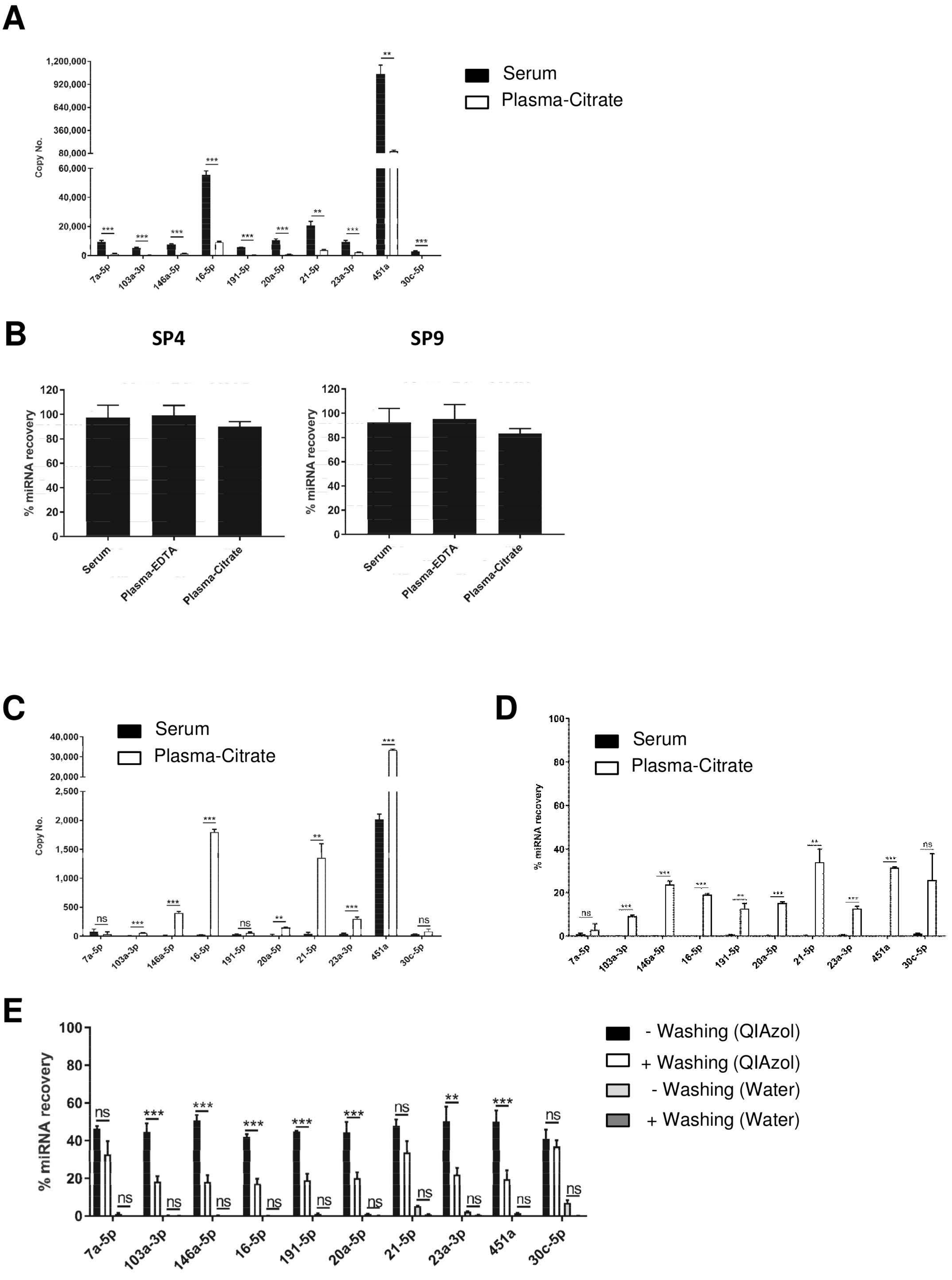

Supplementary Figure 2

A

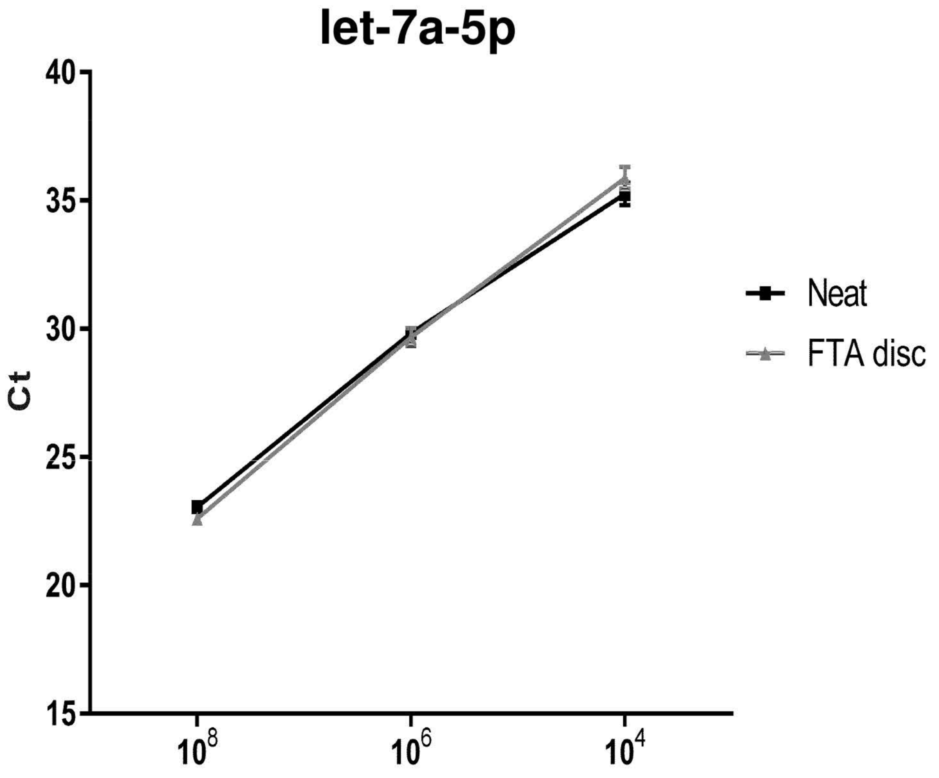

B

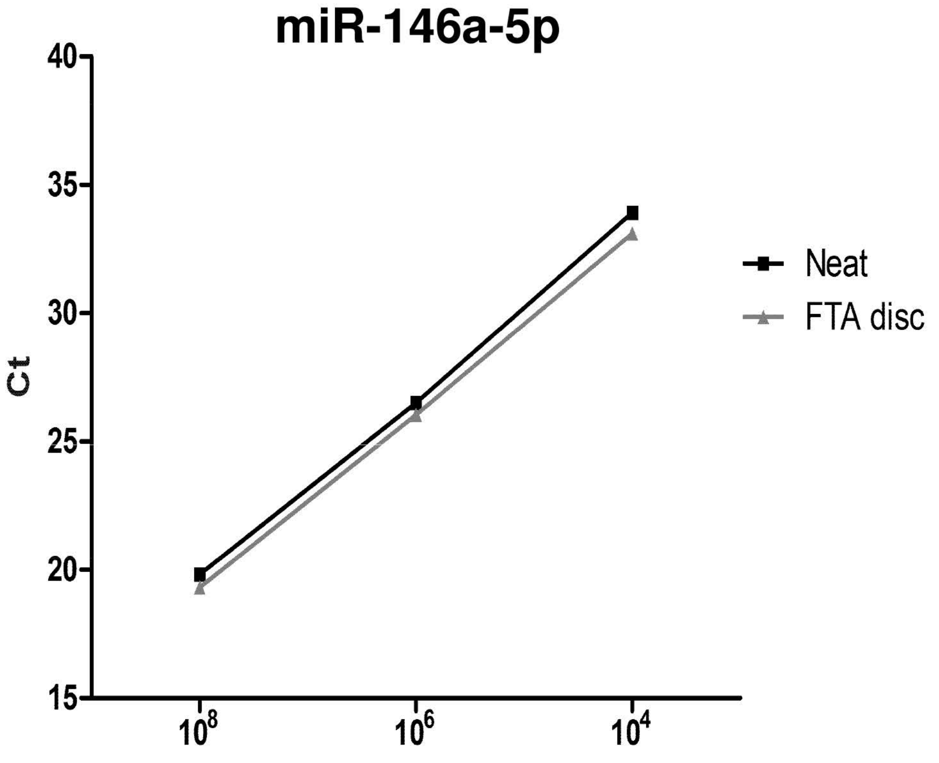

Supplementary Figure 3

A

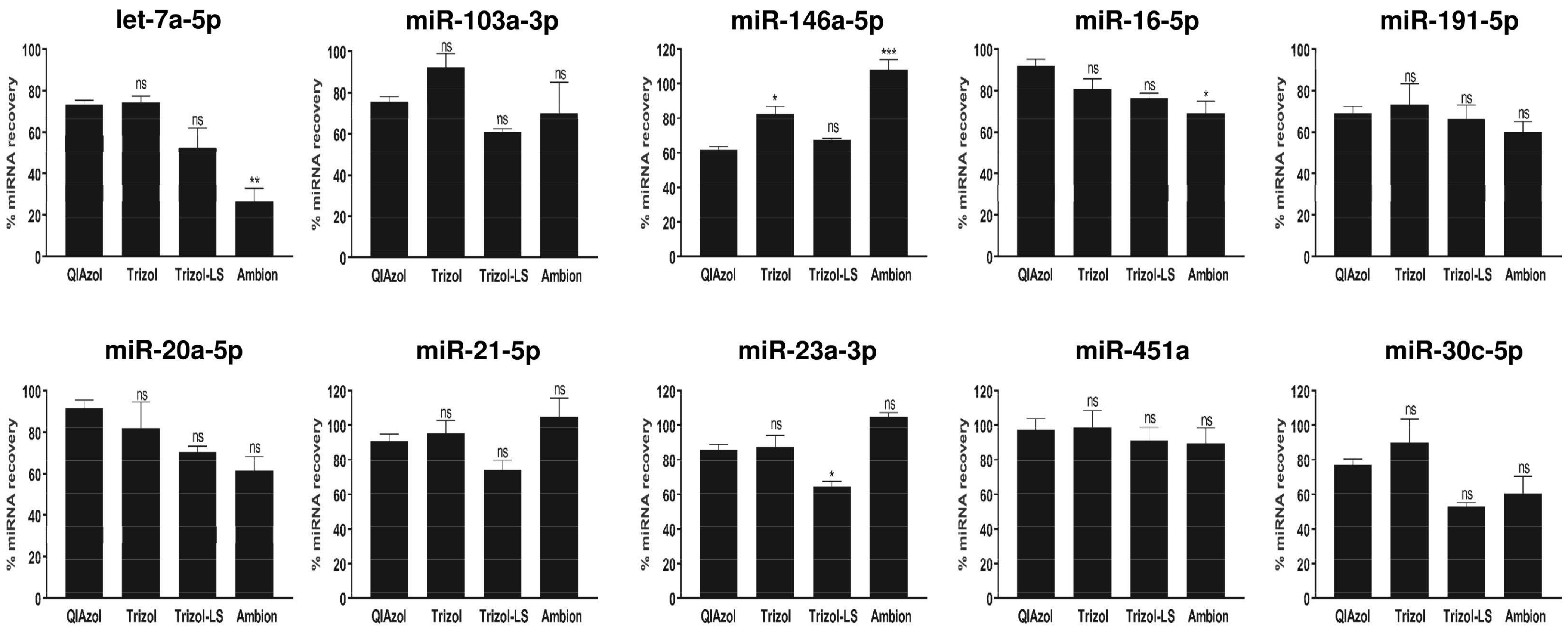

B

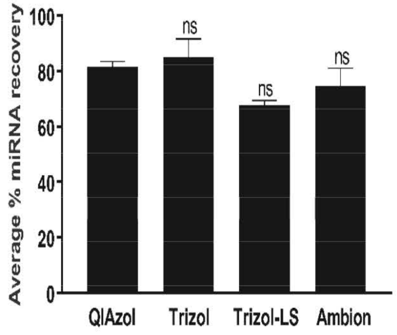

Supplementary Figure 4

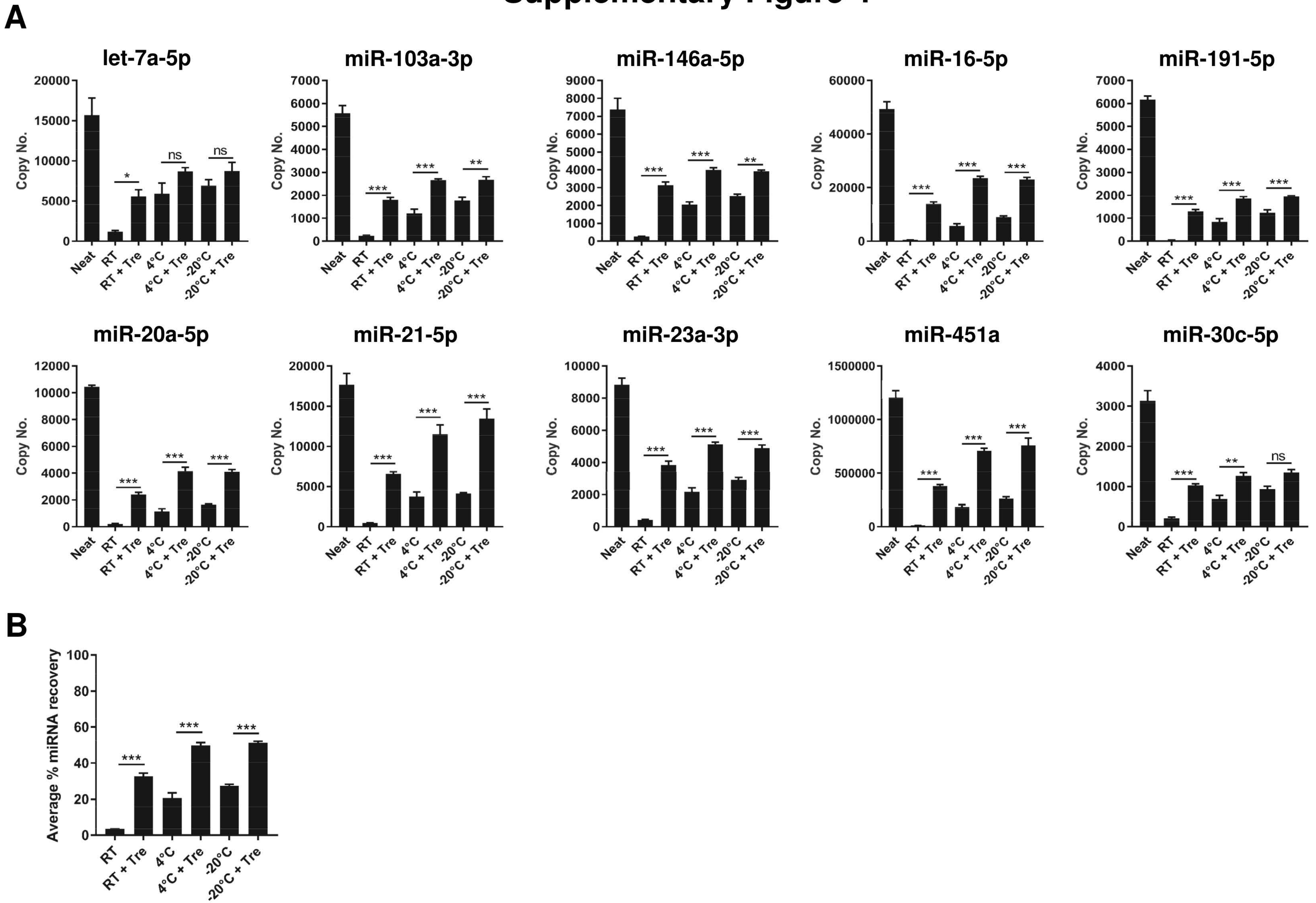

Supplementary Figure 5

A

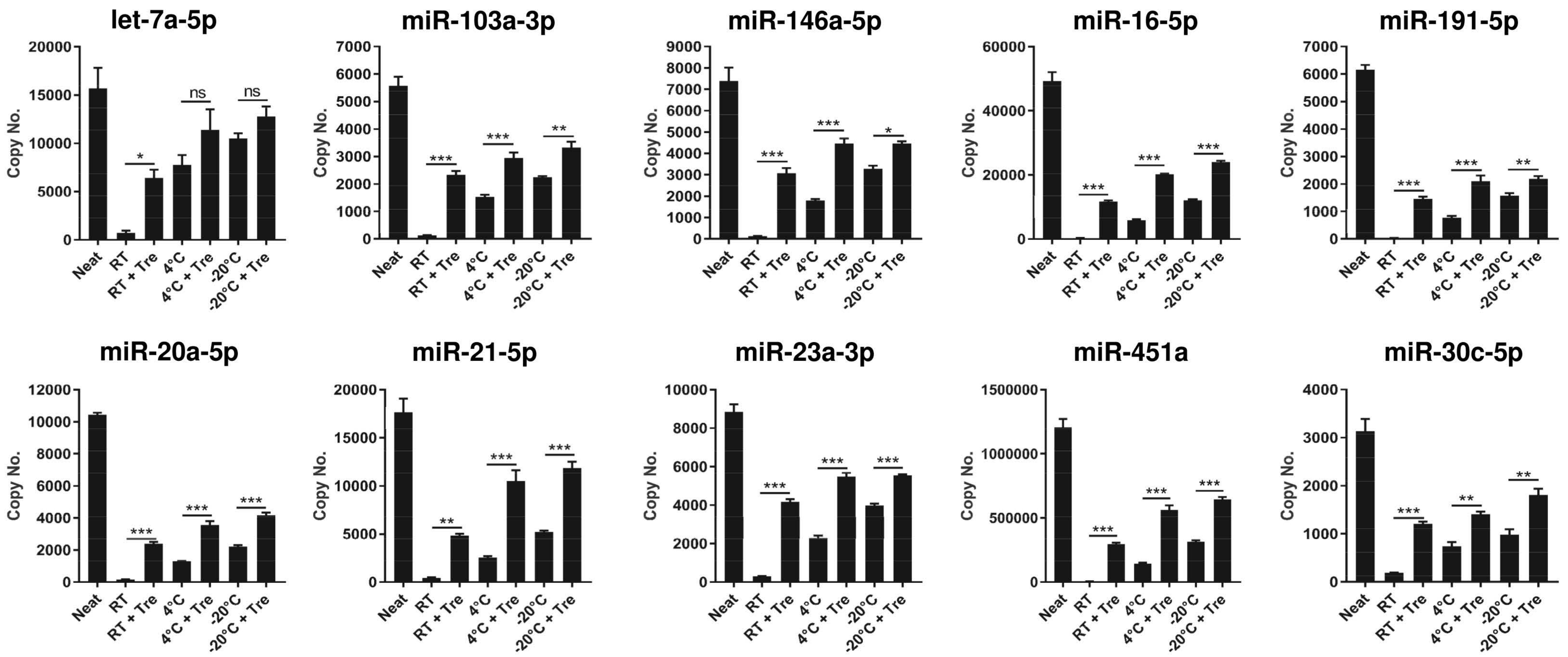

B

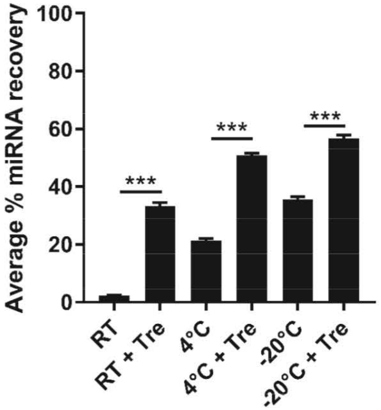

Supplementary Figure 6

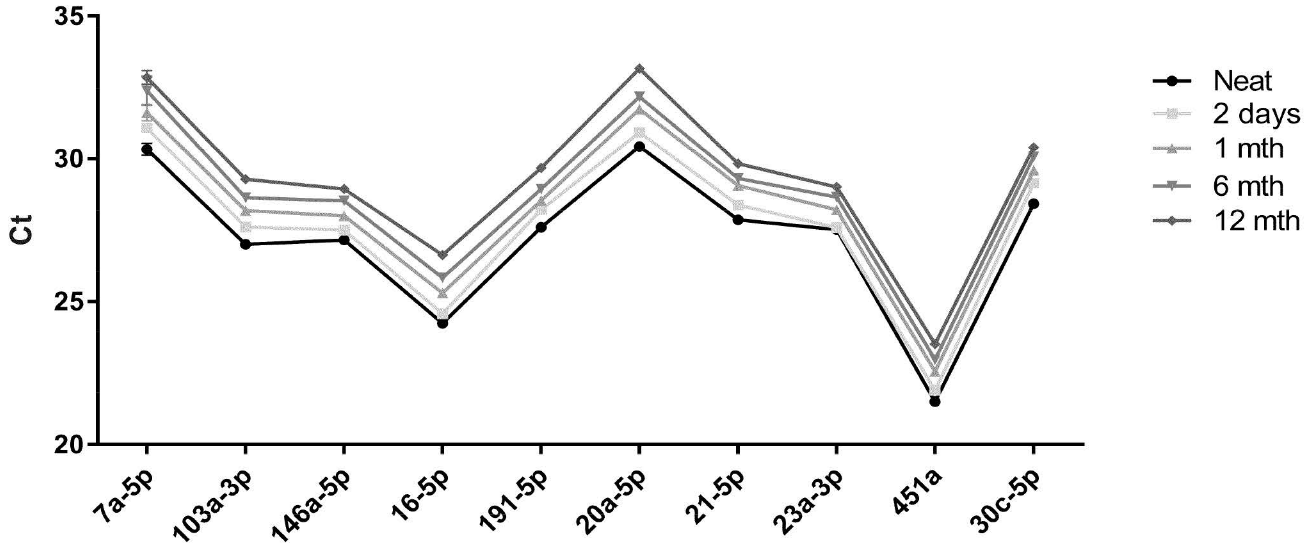

Supplement: Supplementary file 1 — Supplementary Information [file 41598_2017_16960_MOESM1_ESM.pdf]
